# Supplementary material for: Are carbon emissions trading and green financial instruments synergistic? -Comprehensive quantitative research based on content analysis
Source: PLoS One. 2024 Mar 7;19(3):e0298601. doi: 10.1371/journal.pone.0298601 (PMC10919601; doi:10.1371/journal.pone.0298601)
Supplement: S1 Data — (ZIP) [file pone.0298601.s001.zip › Data/地级市Z_F工作报告.zip!/╣≤╓▌/╣≤╤⌠╩╨/└┤╫╘╣1⁄2╓┌║┼í╛┬φ┐╦╩2╛▌═°í┐_2012.docx]

各位代表：

现在，我代表市人民政府，向大会作政府工作报告，请予审议，并请市政协委员、列席人员提出意见和建议。

一、过去五年政府工作回顾

五年来，全市人民在省委、省政府和市委的正确领导下，牢记“作表率、走前列、做贡献”的使命，坚定不移走科学发展路、建生态文明市，圆满完成了市十二届人民政府确定的各项目标任务。

（一）这五年，是发展速度明显加快、经济实力不断增强的五年。牢牢把握贵阳发展阶段性特征，扭住经济建设中心不动摇，加速发展、加快转型，经济发展呈现出速度加快、效益提高、排位前移的良好局面。2006年至2011年，全市生产总值从617.24亿元增至1383.07亿元，年均增长14.7%；人均GDP从15731元提高到31712元；财政总收入从157. 58亿元增至401.36亿元，年均增长20.6%；一般预算收入从61.58亿元增至187.14亿元，年均增长24.9%；全社会固定资产投资从413.36亿元增至1600.59亿元，年均增长31.1%；社会消费品零售总额从234.96亿元增至584.33亿元，年均增长20%。《全球城市竞争力报告（2009—2010）》显示，在全球500个城市中，贵阳市综合竞争力提升了42位，提升速度排列第4位。

（二）这五年，是经济结构不断优化、产业效益大幅提升的五年。三次产业结构中第三产业比重达53%；民营经济增加值占全市生产总值的比重达44%。一是工业发展明显加快。大力实施“工业强市”战略，工业经济呈现投资大、成长快、效益好的局面。规划建设十大产业园区，其中，升格为国家级新型工业化产业示范基地1个、省级示范基地3个、省级开发区4个，全市园区的产业承接力不断提升。开工建设了首钢贵钢新特材料、贵州广铝年产80万吨氧化铝及其深加工等投资规模在5000万元以上的工业项目96个，累计完成工业投资550亿元。通过抓存量、扩增量，全市规模以上工业总产值从669.65亿元增至1436.62亿元、年均增长15.1%，增加值从228.91亿元增至365.23亿元、年均增长14.3%；工业综合经济效益指数从173.65提高到226.01，工业税收占税收总收入的48%。二是现代服务业发展快速推进。发挥省会城市综合优势，加快推进服务业发展，被列入首批“国家服务业综合改革试点城市”。整体提升南江大峡谷、青岩古镇等9个国家4A级景区，成功打造“爽爽的贵阳·中国避暑之都”、“温泉之城”等旅游品牌，旅游人数达到5250.42万人次、旅游总收入达到612.37亿元，年均分别增长23.1%、48.6%。“中国夏季会展名城”品牌效应初显，2011年共举办会展382个，实现综合经济效益7.2亿元。城市金融要素加快聚集，贵阳市商业银行更名为贵阳银行，成功组建贵阳农村商业银行，引进花旗、中信、招商、浦发等一批国内外金融机构，2个村镇银行、43个小额贷款公司、116个融资性担保公司挂牌营业；上市公司发展到13家。全市金融机构人民币存、贷款余额分别从1494.17亿元、1202.85亿元增至3603.65亿元、3012.86亿元，年均分别增长19.3%、20.2%。改貌货运中心站、石板农产品物流园建成营运，二戈寨物流园、西南国际物流中心等项目加快建设，服务全省、辐射周边的商贸流通服务体系正在形成。全市服务业带动社会就业120多万人，对经济增长的贡献率从42.4%提高到48.3%。三是农业产业发展优化提升。围绕农业增效、农民增收、农村发展，大力发展生态农业，推进农业结构调整。市级累计投入财政性农业产业发展资金5.2亿元，是“十五”时期的2.5倍；认证无公害农产品产地604个，面积101.15万亩，经济作物占农作物的比重达60%；发展规模养殖基地1960个，畜产品年产量达到24.4万吨；培育市级以上农业龙头企业113家，带动农户10万户，加工产值突破200亿元，是2006年的2.5倍；农业产业化经营对农民收入的贡献率达20%以上。

（三）这五年，是城乡建设加快推进、环境面貌巨大变化的五年。坚持规划引领、建管并重、功能配套、要素统筹，大力实施城镇化带动战略，推进城乡一体化发展。一是城乡规划体系不断完善。编制了《贵阳市城市总体规划（2009—2020年）》、《贵阳市土地利用总体规划（2006—2020年）》，完成了中心城区控规、重点地区和主要节点的城市设计、77个专业规划以及一市三县城市总规和控规，实现了中心集镇和重点村庄规划全覆盖。二是城市空间大幅拓展。坚持以路网引领城市发展，“三环十六射”城市骨干路网基本建成，完成投资330多亿元；市域快速铁路和城市轨道交通开工建设，完成投资105亿元。城市建成区面积从132平方公里扩大到230平方公里，中心城区规划面积扩展到1230平方公里，城镇化率提高到69.2%。进一步优化功能布局，把中心城区的客车站搬到金阳客运站和客运东站，花溪大道沿线汽贸市场搬到孟关国际汽贸城，五里冲农副产品批发市场搬到石板农产品物流园，城区“内疏外拓”取得重大进展。三是城市服务功能和管理水平进一步提升。国际会展中心、奥体中心体育场、观山湖公园、十里河滩湿地公园、筑城广场等城市精品工程投入使用，世纪城、温泉新城等大型城市综合体初具规模，国际金融中心、花溪大学城、多彩贵州城、城乡规划展览馆等重要城市配套功能项目开工建设。供电、供水、供气及污水处理、公共交通等公用事业加快发展，城市综合服务功能进一步增强。深入开展“整脏治乱”、“三创一办”等活动，建成数字化城市管理系统，采取公交优先、错时上下班、专段号牌管理、尾号限行等综合措施缓解交通拥堵，对违法建筑、非法营运等实施依法整治，对户外广告、渣土运输、占道经营实行规范管理，城市人居环境不断优化。四是城乡基础设施明显改善。推进彭家湾、渔安安井等26个棚户区、城中村改造，实施了中心城区综合管网入地工程，改造农贸市场177个，建成市政道路913公里。配合抓好贵阳至广州、成都、重庆、昆明、长沙等快速铁路、客运专线建设，龙洞堡机场二期扩建工程和开阳港、息烽港建设加快推进。累计投入县乡交通建设资金51.6亿元，实现县际高速公路通达率90%、通乡油路率100%、通村油路率83%。累计投入水利建设资金近10亿元，实施了6大类水利项目，人均有效灌溉面积达0.57亩，90%以上的农村人口基本解决饮水困难。完成村庄整治243个，农村危房改造6.72万户，新建大中型沼气池215口、户用沼气池13.55万口，农村广播电视户通率达90%以上，农电网改造全面完成，群众生产生活条件明显改善。

（四）这五年，是环境保护从严有力、生态优势更加凸显的五年。坚持以生态文明理念引领发展，以最严厉的措施保护青山绿水。组建两湖一库管理局，成立全国首家环保法庭和审判庭，制定了全国首部生态文明建设的地方性法规，投入生态补偿资金5亿多元。深入开展“依法治理两湖一库，确保市民饮水安全”、“森林保卫战”、“除尘降噪”等专项行动，贵阳市民的“三口水缸”水质稳定在Ⅲ类以上；城市污水处理率达95.2%，生活垃圾无害化处理率达93%以上，市区空气质量持续改善；完成155平方公里石漠化治理、采石迹地恢复，森林覆盖率从34.7%提高到42.3%；节能减排目标任务全面完成。成功举办三届生态文明贵阳会议。与联合国气候组织合作开展的“千村计划”项目，在联合国气候变化大会上被评为最佳案例之一。生态文明理念深入人心，一大批生态文明社区、生态文明企业、生态文明学校在城乡涌现，被确定为全国生态文明试点城市和节能减排试点城市，被评为国家森林城市、国家园林城市、全国绿化模范城市、国家节水型城市和全国十大低碳城市，“生态”已成为这座城市的靓丽名片!

（五）这五年，是改革迈出坚实步伐、开放水平持续提高的五年。坚持激活内力与借助外力相结合，围绕破解发展难题推改革、立足提升发展速度促开放，全市发展动力不断提升，发展活力不断增强。一是重点领域改革不断深化。统筹推进财政体制改革、行政审批制度改革、国有企业改革和市属经营性事业单位改制以及农村综合改革。625项市级行政审批事项核减到93项，成为行政审批事项最少的省会城市之一；12家市级投融资公司筹集建设资金504亿元，市场化融资取得重大成果；完成国企改革211家，事业单位改制21家；集体林权制度改革全面完成。二是外向型经济发展步伐加快。共与国内外61个大中城市开通航线80条，与日本惠庭市、新西兰北帕默斯顿市、美国沃斯堡市等友好城市交流合作更加紧密。进出口总额从11.19亿美元增至37.69亿美元，年均增长27.5%；实际直接利用外资从0.7亿美元增至2.79亿美元，年均增长31.8%。累计引进内资项目8208个，实际到位资金2027.2亿元。务实高效、开放创新的发展环境进一步优化提升。

（六）这五年，是社会事业全面进步、幸福指数不断提升的五年。坚持民生优先，着力解决事关群众切身利益的民生问题。各级财政累计投入民生资金509.29亿元，占财政一般预算支出的55%以上；城市居民人均可支配收入达19420元，农民人均纯收入达7381元，年均分别增长11.6%、16.5%；贫困发生率从10.4%下降到7.3%；市民幸福指数提升到89.19。累计新增城镇就业30.17万人，城镇登记失业率控制在3.29%；积极探索“政企并力、建储并举、公廉并轨、租补并行”的保障房供给新模式，4.3万户家庭纳入住房保障；养老、医疗、失业、工伤、生育五大保险征缴扩面；13万城乡低收入人口纳入最低生活保障；新农合覆盖率达97.6%，城镇职工和城镇居民基本医疗参保率分别达91.6%、83.8%，城乡保障体系不断完善。“两基”顺利通过“国检”，学区化改革有序推进，教育均衡化发展水平逐年提高。连续四次被评为“全国社会治安综合治理优秀城市”，获得“长安杯”，群众安全感从2007年的70.8%上升到84.3%。文化、体育、科技、计生等事业长足发展，全面进步，连续五届被评为全国科技进步考核先进城市，获得全国双拥模范城、全国民族团结进步先进集体、法制宣传教育先进城市、中国舞蹈之都、全国人口和计划生育综合改革示范市、国家知识产权工作示范城市等称号。在过去五年中，国防教育、宗教、移民、史志、档案、侨务、对台、气象、老龄、慈善、残疾人、红十字、工青妇等工作全面发展，社会组织在社会事业中的积极作用有效发挥!

过去五年，我们坚持不懈地加强政府自身建设。先后成立市县两级政务服务中心，大力推行简政放权、并联审批、限时办结、首问责任等制度，优化服务方式，增强公共服务能力。完善重大事项社会听证、专家咨询和公示制度，全面推行网上政务公开和办事，“百姓—书记市长交流台”来件初始回复率达到100%。坚持依法行政，自觉接受人大法律监督、工作监督和政协民主监督，办理人大代表建议1056件、政协提案2517件，2011年满意率均达100%。加强政府廉政建设，强化审计监督和行政监察，坚决纠正部门和行业不正之风、严肃查处一批违纪违法案件，政务环境不断优化。过去五年，是我们在经济社会发展道路上迈出坚实步伐、取得可喜成就的五年!

刚刚过去的2011年，我们唱响“主基调”，主攻“主战略”，牢记为人民谋幸福的宗旨，更好更快推动经济社会发展，实现了“十二五”良好开局，为本届政府工作划上了圆满句号，为下届政府工作奠定了良好基础。

一年来，我们奋力加速、领跑“十二五”。全市生产总值、全社会固定资产投资、规模以上工业增加值、社会消费品零售总额、财政总收入、一般预算收入、城市居民人均可支配收入、农民人均纯收入分别比上年增长17.1%、57.1%、23.4%、20.5%、31.7%、37.3%、17%、23.5%，多项经济指标增速在全国省会城市中位居前列。在全省经济发展增比进位综合排位中，位居第1名。全市人民在“十二五”第一棒奋力加速，跑出了令人鼓舞的好成绩!

一年来，我们协力争先、提振精气神。432万贵阳人民团结协作，46万绿丝带志愿者辛勤付出，广大党员干部奋力拼搏，全力冲刺“三创一办”，凝聚和提振了“知行合一，协力争先”的贵阳精神，成功、圆满、精彩地协办了第九届全国少数民族传统体育运动会，被省委、省政府授予唯一的“特别贡献奖”；双双荣膺“全国文明城市”、“国家卫生城市”称号。生态文明贵阳会议、贵州首届国际车展、首届中国（贵州）国际酒类博览会暨投洽会、第五届全国工商联执委会暨全国民营企业助推贵州发展大会、筑城广场万人迎新活动等成功在贵阳举办。通过节会聚力、活动扬势，塑起城市人文新坐标，提振了贵阳协力争先的精气神。

一年来，我们竭力创新、突破新领域。瞄准世界前沿，主攻科技创新，居世界领先水平的皓天光电LED蓝宝石衬底材料成功下线，振华年产3000吨锂离子电池正极材料全面达产，中航工业凯阳航空发动机维修基地试车台点火成功，海信平板模组核心部件实现本地化生产，全省首家在创业板上市的互联网企业朗玛公司通过中国证监会上市核准。全市高新技术企业达到107家，占全省的69.9%，高新技术产业增加值占全市规模以上工业增加值的32%。我们积极推进基层管理体制改革，变四级管理为三级管理，实现服务职能下移；组建群众工作委员会和群众工作中心，畅通群众诉求渠道、变“中转站”为“终点站”；成立和谐促进会，增强社会矛盾化解合力；创新流动人口管理服务，开展“城市绿卡”发放试点工作，让新来这座城市的“客人”享受同城待遇。我们在创新中不断实现新的突破，在突破中不断积累新的经验。

一年来，我们致力跨越、奠定新基石。把开局之年作为基础之年，着眼于新起点上的新跨越，完成1600.59亿元固定资产投资，其中，实施了总投资44.58亿元的交通基础设施项目、3.18亿元的水利基础设施项目、19.3亿元的电力基础设施项目、13亿元的通信基础设施项目，着力提升支撑跨越的硬件基础。完成十大产业园区基础投资215亿元，占园区累计基础投资的87.7%；引进到位产业类项目资金224.74亿元，先后开工建设150个总投资700多亿元的工业项目，全部达产后预计年新增产值1000亿元，着力提升支撑跨越的产业基础。投入资金162亿元，扎实推进“十大民生工程”，多干利民之事，进一步拓宽社会保障覆盖面，筑牢民生网底，着力提升支撑跨越的保障基础。

回顾五年的工作，我们深刻地体会到：必须在市委的坚强领导下，确立把上级精神与本地实际紧密结合的发展思路，并坚持不懈地为之奋斗。这是一条最根本的经验！

总结五年的工作，我们也清醒地看到前进中存在的突出问题，主要表现在：经济总量不大，产业结构不优，转型速度不快，高新技术支撑不足，城乡居民收入水平不高；发展基础还需进一步夯实，综合带动力还需进一步提升，民生保障还需进一步完善，政府职能还需进一步转变，服务意识还需进一步增强，工作效率还需进一步提高等等。对这些问题，我们将采取更加有为的措施认真加以解决，努力让人民群众更加满意！

各位代表，五年的奋进历程充满艰辛，五年的发展成绩来之不易。我们深知，这是省委、省政府和市委正确领导的结果，是在历届市委、市政府打下坚实基础上取得的结果，是市人大、市政协有效监督、大力支持的结果，是全市干部群众努力奋斗、辛勤劳动的结果。在此，我代表市人民政府，向全市人民，向各位人大代表和政协委员，向各民主党派、工商联、人民团体和各界人士，向中央及省在筑单位，向驻筑人民解放军、武警官兵和公安政法干警，向所有关心和支持贵阳建设与发展的各位老领导、老同志和各界朋友，表示衷心的感谢，并致以崇高的敬意!

二、今后五年政府工作的总体要求、奋斗目标和战略重点

今后五年，是贵阳工业化、城镇化、农业现代化发展的攻坚期和加速期，是贵阳全面发力、奋力攀高、阔步领跑的黄金期和关键期。我们将认真贯彻落实国发〔2012〕2号文件精神，牢牢把握新一轮西部大开发、新一轮扶贫开发、东部产业加速向中西部地区转移、黔中经济区战略规划实施等重大战略机遇，树立世界眼光，强化战略思维，乘势而上，奋发有为，加快建设全国生态文明城市、国家创新型城市、西部地区高新技术产业重要基地、区域性商贸物流会展中心、内陆开放型经济示范区、“爽爽贵阳”旅游休闲度假胜地，努力铸就贵阳发展新辉煌。

今后五年政府工作总体要求：高举中国特色社会主义伟大旗帜，以邓小平理论和“三个代表”重要思想为指导，深入贯彻落实科学发展观，在省委、省政府和市委的坚强领导下，认真贯彻落实市第九次党代会精神，紧扣“两加一推”主基调，实施“工业强省、城镇化带动”主战略和“三化同步”总要求，“坚持走科学发展路、加快建生态文明市”，继续弘扬“开放创新，团结奋进”的贵州时代精神，“知行合一，协力争先”的贵阳精神，提振信心、提升动力、提质转型、提速发展，当好全省经济社会发展的“火车头”、黔中经济区崛起的“发动机”，更好更快地推进我市经济社会实现新的历史性跨越。

今后五年政府工作奋斗目标：加快建设经济实力更强、生态环境更好、幸福指数更高的生态文明城市，全力实现“一先二超一提升”的目标(提前五年在全省率先实现全面小康；经济总量增长1.4倍以上、超1个省会城市、排到西部第6位、进入第二梯队，经济年均增长17%以上、超约7个省会城市、进入全国前列；市民幸福指数提升3到5个点，达到93左右)。

今后五年政府工作战略重点：坚持六大发展，构建六大体系。

(一)坚持转型发展，构建绿色经济生态体系

围绕建设全国重要能源基地、资源深加工基地、特色轻工业基地、以航天航空为重点的装备制造基地、循环经济示范基地、新材料产业基地、生态低碳城市等目标，坚持产业绿色化、绿色产业化发展理念，把发展绿色经济作为转变经济发展方式的主要途径、作为实现可持续发展的战略取向、作为建设生态文明城市的重要引擎，采取最严格的手段保护生态、用科学的方式推进生产、用绿色的理念倡导消费，正确处理“转”与“赶”、“好”与“快”以及提速增量与降耗减排三大关系，通过科技创新驱动、产业集群联动、市场需求带动、政策扶持推动，集聚综合要素，促进生态资源转变为生态资本、生态优势转化为经济优势，加快构建以生态从严保护、资源深度开发、生产清洁低碳、产业升级高效为主要特征，以绿色农业为基础、绿色工业为支撑、绿色服务业为主导的绿色经济体系，推进绿色经济生态建设，提升城市发展的可持续竞争力。通过五年努力，全市生产总值、财政总收入分别突破3000亿元、1000亿元；服务业增加值占地区生产总值57%以上；科技进步对经济增长的贡献率超过55%；高新技术产业增加值占规模以上工业增加值的比重超过38%；单位GDP能耗降低16%，主要污染物排放总量减少8—14%。

(二)坚持统筹发展，构建宜居城镇生态体系

按照“融入国际化、实现现代化、体现人文化、突出生态化”和“世界眼光、国内一流、贵阳特色”的要求，从打造新的战略支点和培育新的增长极出发，超前谋划黔中经济区核心区发展，高起点规划、高标准建设“三区五城五带”（贵安新区、双龙新区、北部新区；百花生态新城、花溪生态新城、天河潭新城、龙洞堡新城、北部工业新城；二环四路城市带、贵阳至遵义城市带、贵阳至安顺城市带、贵阳至毕节城市带、贵阳至凯里及都匀城市带），进一步优化城市空间布局、完善配套服务功能、推进产业集群发展、提高区域间快速通达能力，增强省会城市在全省的辐射带动功能。通过以城带乡、产城互动、城乡一体、统筹发展，着力在空间形态、交通构架、服务网络、产业布局、政策创新等方面打破城乡二元结构，加快构建空间优化、要素聚集、产业发展、环境优美、交通便利、生活舒适、文明安全、人文和谐的宜居城镇体系，推进宜居城镇生态建设，使城镇让生活更加美好。通过五年努力，中心城区建设用地规模达到380平方公里，新增城镇人口150万人，城镇化率达到75%以上，城镇经济占经济总量的比重达90%；城市建成区绿化率达到45%以上；城市棚户区、城中村改造率达到80%以上，中心村村庄整治覆盖率达到60%以上；市民公交出行分担率超过45%，行政村通班车率达到90%。

（三）坚持包容发展，构建自强文化生态体系

有容乃大，自强则立。站在新的历史起点，“贵阳当自强”正奏响最强音。这种自强来自奋勇攀高的进取心，来自敢打敢拼的自信心，来自对这座城市的认同感和自豪感。围绕建设全国文化交流重要平台、全省文化产业核心区的目标，坚持兼容并蓄、博采众长的开放包容理念，大力实施文化强基、文化惠民、文化保护、文化产业、文化诚信和文化人才“六大文化工程”建设，加快构建以多样性文化和谐共生、原生态民族文化繁荣发展为特色，以培养文化自觉、文化自信、文化自强为核心，以覆盖城乡、结构合理、功能健全、实用高效为基础的公共文化服务体系，不断丰富“知”与“行”的内涵，推进自强文化生态建设，提升城市软实力。通过五年努力，市民文明程度大幅提升，文化氛围更加浓厚，文化业态更加丰富，文化产业增加值占生产总值的5%以上；文化信息资源共享工程覆盖100％行政村、数字农家书屋覆盖100%行政村、数字电影放映设备覆盖100%乡镇，广播电视入户率达到95%以上。

（四）坚持持续发展，构建友好自然生态体系

注重用生态化理念发展产业、用产业化理念建设生态，正确处理环境保护与经济发展的关系，更加敬畏自然，更加尊重自然，更加善待自然，深入实施治水、护林、净气、保土工程，大力节能、节水、节地、节材，对资源实行有序开发、有偿利用，注重生物多样性的保护与建立，加快构建以水生态、林业生态、气候生态、土壤生态和生物多样性系统为支撑，以天更蓝、地更绿、水更清、空气更清新、环境更优美、人与自然和谐相处为目标的可持续自然生态体系，推进友好自然生态建设，让我们生存的家园更加美丽。通过五年努力，全市水源地一级保护区水质控制在Ⅱ类以上，二级保护区水质控制在Ⅲ类以上，集中式饮用水源地水质达标率100%；森林覆盖率达到45%，中心城区空气质量稳定提升；城市污水处理率达到98%，生活垃圾无害化处理率达到100%；农村污水处理率达到30％以上，生活垃圾定点存放清运率达到100％。

（五）坚持共享发展，构建和谐社会生态体系

坚持发展为了人民、发展成果由人民共享，更加注重人的全面发展和经济社会的协调发展，大力实施民生工程，推进文化生活大众化、社会保障普惠化、公共服务均等化、社会管理创新化、社会关怀人文化，着力构建学有所教、劳有所得、病有所医、老有所养、住有所居、业有其岗、保障完善、管理有序的和谐社会体系，加快和谐社会生态建设，让人民群众在这座城市生活得更有尊严、更有品质、更加阳光、更加幸福。通过五年的努力，全市财政民生支出比重提高到64%；城乡居民收入分别达到39000元、18000元，农村贫困群众全面脱贫；城镇人均住房建筑面积30平方米以上；城镇登记失业率控制在4.5%以内；建立覆盖城乡的社会保障体系和公共服务体系，城镇居民最低生活保障率达到98%以上，每千人医生数超过3人，大学入学率超过20%；群众安全感超过90%，市民幸福指数提升到93左右。

（六）坚持改革发展，构建协调政治生态体系

始终坚持党的领导，高举“发展、团结、奋斗”的旗帜，秉持为人民谋幸福的理念和一切为了人民的价值取向，大胆改革和摒弃不适宜“两加一推”主基调、不符合“坚持走科学发展路，加快建生态文明市”总路径、不适应“一先二超一提升”总目标要求的思想观念、工作作风、工作方法和体制机制，进一步推进行政管理体制改革、政府职能转变和公务员队伍建设，加快提升政府系统的发展创造力、干事凝聚力、决策公信力、工作执行力和队伍战斗力，进一步解放思想、开拓创新、廉洁从政，积极营造保护创新者、支持改革者、鼓励发展者、宽容失误者，和衷共济、共同担当的干事创业环境，构建团结协作、务实高效、公正透明、诚实守信、清正廉洁、勤政为民的行政服务体系，促进协调政治生态建设，为在全省率先实现全面小康提供重要政治保障。“一报告两评议”群众测评满意率逐年提高；破坏投资环境案件和公务员队伍违纪违法案件逐年下降；群众对政府工作的满意度大幅提升！

三、今年的目标任务和工作重点

今年是新一届市委、市政府履职的第一年，是实施“十二五”规划承上启下的关键之年，是向全面实现小康社会发起主攻之年。做好今年各项工作，意义重大。今年经济社会发展的主要预期目标是：生产总值增长17%（一产增长6%，二产增长20.6%，三产增长15%）；财政总收入增长18%，一般预算收入增长18%；全社会固定资产投资增长55%；社会消费品零售总额增长18%；实际直接利用外资增长30%；外贸进出口总额增长17%；城市居民人均可支配收入增长16%，农民人均纯收入增长18%；城镇登记失业率控制在4.5%以内；城镇化率增加1个百分点；人口自然增长率控制在5‰以内；居民消费价格指数控制在国家调控目标范围内；单位生产总值综合能耗、主要污染物排放总量控制在省下达指标内。

我们将以贯彻落实国发〔2012〕2号文件为突破口，按照“稳中求进、提速转型”的总基调、总目标，“稳中求快、快中保好、能快则快、又好又快”的总要求，坚持走科学发展路，加快建生态文明市，突出八大重点、实现八个新突破：

（一）以投资拉动为重点，在发展速度提升上实现新突破

坚持把投资作为扩大内需的主渠道、作为拉动增长的主抓手，下大力抓项目、增投资、提速度，确保固定资产投资完成2480亿元。一是超前谋划项目。紧盯国家投资走向和市场经济走势，特别是国发〔2012〕2号文件中明确的发展定位、战略重点、项目支撑和政策支持，加大项目规划编制力度，加大项目前期经费投入，做深做实基础性工作，谋划一批大项目、好项目，使项目库动态保持在5000亿元以上。二是积极争取项目。坚持内外结合，一方面全力以赴争取上级项目投入，力争更多项目挤入国家和省发展盘子，确保国家、省属项目完成投资280亿元。一方面全方位推进对外招商，突出“世界500强、国内500强企业，现有产业中的上下游延伸和横向配套项目，关联度高的新业态项目”三个重点，坚持“亩均投资强度不高的项目不签，不符合环保要求的项目不签，圈地项目不签”三条原则，创新招商方式，力争引进市外资金增长30%以上。三是高效服务项目。坚持重大项目领导挂牌服务，做到项目审批不到位不放松、不按期开工不放松、不按进度推进不放松、不按要求投入使用不放松。全力破解用地问题。通过向上争取指标和点供直供政策、盘活土地存量、开展城乡建设用地增减挂钩、推进山地整治、低丘缓坡荒滩等未利用地开发利用试点、合理利用废弃矿山采空区土地、节约集约用地等多种措施，提高用地效率，把握用地节奏，缓解用地矛盾。全力破解融资问题。提高财政资金使用效益，集聚社会资本设立产业发展基金和创业投资基金；加强银企合作，扩大金融机构直接融资规模，并优化向农业产业、中小企业、微型企业的投向结构；积极引导民间金融机构发展；助推符合条件的企业上市；力争市级投融资公司基本调整为一般类公司客户，通过发债、信托、中期票据等方式提升融资能力；充分利用资源、资产、资本、技术、服务、预期收益等要素激活融资，鼓励和引导社会资本更广泛地参与项目建设和产业发展，为推进经济自主协调发展创造良好条件和宽松氛围。四是快速推进项目。重点抓好总投资3834.6亿元的173个省级重大工程和重点项目建设，年内完成投资616亿元。积极配合做好贵阳至瓮安高速公路等项目前期工作，力促开工贵阳至惠水、清镇至黔西、厦蓉高速清镇至织金段等高速公路项目。继续配合抓好贵阳至广州、成都、重庆、长沙、昆明等快速铁路建设和龙洞堡机场二期扩建工程。加快推进轻轨、市域快速铁路、开阳港、息烽港、渔洞峡水库等基础设施项目和首钢贵钢新特材料、乙二醇、奇瑞贵阳客车等重大产业项目建设。力争完成基础设施投资744亿元、工业投资840亿元，分别占固定资产投资总量的30%和34%，进一步优化投资结构，发挥投资效益。

（二）以转型升级为重点，在工业做大做强上实现新突破

围绕传统产业高端化、特色产业集团化、高新产业规模化的要求，以推进新型工业化综合配套改革试点为契机，突出产业园区建设、优势产业提升、新兴产业培育三大重点，强化科技支撑、加强运行调度、增强要素保障、注重资源整合，通过盘活存量、做大增量，使规模以上工业总产值增长30%，达到1800亿元。一是加快推进园区建设发展，全面提升产业聚集力和配套力。坚持把园区建设作为承接工业发展的基础支撑、作为产业集聚的重要平台，完善供能站、供能管道、供水管网、电力、通信、标准厂房等基本要素，加快金石产业园中广线等各园区进出路网建设，提高企业入驻条件，完成园区基础设施投资230亿元，产业投资310亿元，力争十大园区工业总产值突破1000亿元。加快国家高新技术产业开发区、国家经济技术开发区的扩区调位规划申报工作，力争麦架—沙文高新技术产业园、息烽循环经济工业园、清镇工业园、修文经济技术开发区、开阳工业园建成国家级新型工业化产业示范基地，推进其它园区向省级产业园区升级。二是加快推进特色优势产业升级扩能，壮大工业经济总量。抢抓“织金—息烽—开阳—瓮安—福泉磷煤化工产业带，清镇—黔西—织金煤电铝、煤电化循环经济示范基地”建设的机遇，以推进煤电钢、煤电磷、煤电铝、煤电化“四个一体化”发展为重点，全面提高资源就地转化率和主导产品本地配套率，促进多产业共生耦合、聚集发展。积极争取开工建设息烽至金沙、清镇至黔西等能源通道项目，积极推进直供电试点，努力构建支撑产业发展的能源保障体系。大力实施“三个一百”工程（确保100个重点工业项目开工、100个已开工项目投产、100个已投产项目达产），使装备制造、磷煤化工、铝及铝加工、现代制药、特色食品、烟草制品等特色优势产业完成产值1400亿元，占规模以上工业总产值75%以上。下大力气培育龙头企业和全国性、国际性品牌，新增2户产值上100亿元企业、10户产值上10亿元企业，培育50户高成长性企业，提升企业市场竞争力。三是加快推进战略性新兴产业培育发展，优化工业经济结构。坚持科技创新驱动，紧盯高端领域和产业发展瓶颈，引进或培育建设一批重点实验室、工程技术研究中心，力争打造3—4个省级产业技术创新联盟，培育一批创新型企业，实施一批科技重大专项和重点项目，大力引进和培养行业领军人才、核心专家、特殊专业技术人才，以优秀的人才队伍、优越的创业环境、优惠的支持政策支撑战略性新兴产业发展。切实抓好中航工业贵阳产业基地、南车集团贵阳新产业基地、振华新材料新能源产业基地、贵阳数字内容产业园、雅洛科技等项目建设，促进皓天光电LED蓝宝石衬底材料（一期）、时代沃顿复合反渗透膜等项目达产增效。加快推进工业化和信息化融合发展，大力引进知名IT企业、数据中心落地贵阳，推进“慧谷”项目的规划实施。千方百计推进新材料、新能源、高端装备制造、生物医药、生物育种、节能环保、光伏产品、电子及新一代信息技术等战略性新兴产业加快发展，实现高新技术产业增加值增长30%以上。

（三）以优化空间布局为重点，在提升城市发展水平上实现新突破

坚持用世界眼光的高度规划城市、用国内一流的标准建设城市、用贵阳特色的要求塑造城市，加快提升城市辐射力、承载力、服务力和形象力。一是突出城市辐射力的提升，加快规划建设“三区五城五带”。为充分发挥省会城市的引擎功能，高标准完成“三区五城五带”的战略策划、总体规划、控制性规划和城市设计，加快编制路网、给排水、绿地、产业发展、功能布局、环境标准等专项规划。坚持“市统筹、区为主、同标准、快建设”原则，切实抓好土地收储、拆迁安置、融资保障、依法控违拆违等工作，强力推进二环四路城市带建设，启动建设贵阳至开阳高速公路，白云至修文、贵阳至遵义城市带主干道一期工程以及党武至湖潮、龙洞堡至龙里等城市干道，建成金阳至清镇市政干道，通过路网引领城市发展，优化城市空间布局，拉动“一市三县”同城化发展，不断实现黔中经济区城市群之间的高效连接。二是突出城市承载力的提升，加强城市基础设施建设。优化城市路网结构。完成盐沙路、富源南路、东站路一期工程、桐荫北路建设；实施主城区路网加密工程，畅通“微循环”；加快市域快速铁路和轻轨1号线建设，做好后续项目前期工作；启动建设地下人防通道工程，建设一批人行过街天桥，合理分解人车出行。完善城市配套基础设施。加快自来水厂、污水处理厂、接污管网、垃圾焚烧发电等项目建设，新建、改建垃圾中转站12座、公厕15座；加快推进“三网融合”，启动物联网基础设施规划建设，运用新兴网络和现代信息技术，积极打造创新型智慧城市。优化提升主城区。按照“规划一片，改造一片，提升一片”的要求，启动推进金桥地块、九华大院地块、白金片区、东山片区、五里冲片区、公园路片区等棚户区、城中村改造项目；出台优惠政策，支持城市中心区发展“飞地工业经济”，支持城区现有工业企业“退二进三”、“退工进商”；引导主城区建材、家具等大型市场向二环四路城市带周边疏解布局；大力实施城区综合整治，打造文明示范小区，实现城区环境点面结合，整体提升。三是突出城市服务力的提升，完善城市综合配套功能。继续推进客运站、货运站和公交专用道、停车场建设，优化公交站点，完善道路交通智能监控指挥系统，加大对交通堵点乱点和非法营运整治力度，进一步缓解“出行难、公交难、打车难、停车难”现状。加快星级酒店建设和市民公共文化娱乐设施、城市山体公园建设，充分发挥筑城广场“城市客厅”的功能作用。抓好奥特莱斯、西南国际商贸物流城、苏宁广场、贵州文化广场等一批城市综合体建设，在城市新聚集区引驻一批大型超市、连锁店，提升城市商、住、娱、购等综合功能。切实增强金阳新区综合要素组配力和现代产业服务功能，致力打造市级文化体育中心、西南商业金融中心和会展中心，加快建设生态型、园林式现代化新城。四是突出城市形象力的提升，提高城市科学管理水平。重点打好“饮用水源地、城乡结合部、背街小巷、小区院落、农贸市场、建筑工地、公共广场、公共厕所”八大整治战役，深入实施“蓝天、碧水、绿地、亮化、宁静、畅通、文明、安全”八大工程，继续加大工作力度，创新工作机制，切实巩固全国文明城市、国家卫生城市创建成果，积极推进创建国家环境保护模范城市工作，推动城市管理向管精、管实、管长效转变。进一步加强城市品牌营销，提升对外形象，让贵阳成为更加美丽的生态宜居之城、吸金创业之地。

（四）以优化资源配置为重点，在现代服务业发展上实现新突破

坚持把服务业作为就业的最大容纳器、作为产业融合发展的粘合剂、作为经济结构调整的重要突破口，充分发挥贵阳气候、文化、环境、区位等综合优势，着力在提升速度、优化结构、丰富业态上下功夫，促进现代服务业大突破、大提高、大发展。一是推进旅游业大发展。围绕打造“爽爽贵阳”旅游休闲度假胜地和世界知名、国内一流旅游目的地、旅游集散中心的目标，进一步加强旅游基础设施及服务功能设施建设，推进旅游行业标准化建设，优化提升“吃、住、行、游、购、娱”旅游要素功能。有序规划建设生态型多梯度高原运动训练基地和山地户外体育旅游休闲基地；加快多彩贵州城、南明河城市流域旅游文化街区、清镇国际旅游休闲体育度假区、美食文化街、游客服务中心等项目建设；推进天河潭、青岩古镇、南江大峡谷、桃源河等景区创建国家5A级旅游景区工作；精心打造“爽爽贵阳”交响音乐会、息烽红色记忆等文化旅游精品节目；新开2—3条国内航线、1—2条国际航线或加密航班。通过发挥集散功能，聚集省内及周边知名旅游产品的联动效应，精心策划、高端展示，筹办好第六届旅游产业发展大会、国际山地自行车邀请赛、中超足球联赛以及避暑季、温泉季等系列活动，加快旅游产业发展，实现旅游人次和旅游收入分别增长20%、30%。二是推进会展业大发展。以建设中国夏季会展名城为目标，制定并实施会展业发展规划，以项目品牌化、运作市场化、服务专业化为方向，提升生态文明贵阳会议、国际酒类博览会、亚洲青年动漫大赛等会展品牌影响力，完善国际会展中心等平台服务功能，抓好新的会展资源开发，力争举办100个以上大型展会，促进会展与产业互动融合，实现展会综合经济效益9亿元以上。三是促进金融业大发展。加快推进城市金融要素的聚集，在现有27家商业银行的基础上，实现民生银行、兴业银行、光大银行等驻筑分支机构挂牌开业。加快推进贵阳银行上市和跨区域经营，支持贵阳农村商业银行等做大做强。加快国际金融中心建设，积极鼓励和引导各类金融、证券、保险机构入驻。大力发展小额贷款公司、融资性担保公司、村镇银行、邮政储蓄银行、农村合作金融机构，激活民间金融市场，更好地服务经济发展。四是促进商贸物流业大发展。用好用活全国流通领域物流示范城市政策，加快打造西部区域物流中心。重点推进改貌、清镇、扎佐、息烽九寨物流园区和龙洞堡空港物流中心、花溪工业商贸物流园等建设，支持第三方物流加快发展，新增3A级以上物流企业4家。加快中大国际、保利国际等大型商业综合体建设，优化城市传统商业街区业态组合。支持星力、国贸等大型零售企业开展多元化经营，打造商业“航母”；支持建设现代化的中药材批发市场和酒类、茶叶交易市场。五是加快新兴服务业发展。适应新型业态多样化发展需求，积极支持和加快科学技术、信息产业、文化创意、服务外包、医疗服务、物联网以及研发设计、检测检验、科技成果转化、电子商务、生物技术等高技术服务业发展，提升省会城市的现代化发展能级。

（五）以“三农”工作为重点，在城乡统筹发展上实现新突破

认真贯彻落实中央农村工作会议精神，按照“三化同步”的要求，以工业化提升农业，促进农业向工业转换；以城镇化统筹农村，促进农村向城市转化；以市场化引导农民，促进农民向市民转变，努力推进城乡统筹发展。一是以增加农民收入为着力点，加快农业产业结构调整。按照“立足农业、跳出农业抓农业产业调整，立足农村、跳出农村抓农村经济发展，立足农民、跳出农民抓农民增收致富”的思路，在农业规模化、市场化、产业化发展上实现重大突破，加快建设山区现代化农业示范区。坚持因地制宜布局、集中连片规划、整乡整片推进，集约化发展肉、禽、蛋、奶等现代生态畜牧业，规模化种植蔬菜、果树、中药材、茶叶、花卉苗木等经济作物，在“三环”以内全面取消传统农作物种植，全市新增经济作物40万亩，粮经比由4：6调整到3：7。加大农村土地流转力度，强化农业实用技术推广，提高生产水平。加快推进农业产业化经营，重点扶持老干妈、黔五福、好一多等农业龙头企业40家，农民专业合作组织100家，带动农户5万户、发展基地10万亩，使农产品加工转化率提高2个百分点。认真落实“菜篮子”市长、区长负责制，发展万亩蔬菜专业乡镇（村）12个，建设蔬菜标准示范园20个，完成蔬菜播面155万亩（次）。二是以提高农业生产力为着力点，加强农业基础设施建设。掀起农田水利建设新高潮，完成基本农田基础设施建设3万亩，加快推进息烽两岔河等5座中型水库的前期工作，开工建设花溪红岩、开阳那卡河等中型水库和乌当金家箐、清镇席关等7座小型骨干水库工程，完成修文后洞等14座病险水库除险加固、青岩河等4条中小河流治理，新增解决农村23.67万人饮水安全，治理水土流失面积150平方公里，完成清洁小流域治理试点6平方公里。因地制宜推进山区农业机械化进程，不断提升现代农业装备水平。加快建立完善农业气象服务体系和农村气象灾害防御体系，切实发挥气象保障功能。三是以改善农村生活环境为着力点，提高村镇建设水平。完成739个行政村村庄规划编制工作，实现城乡规划全覆盖。实施农村“通畅工程”，改造农村公路200公里，新建串户路1000公里，建制村公路硬化率和通班车率分别达到84.5%、87%。整合资金，在高速（高等级）公路沿线，园区景区周边打造30个产业型、商贸服务型、旅游观光型特色小城镇，完善功能、提升品位，增强小城镇承接城乡统筹发展的纽带功能。纵深推进村庄综合整治，使全市33%的村庄达到“布局合理、设施配套、环境整洁、特色明显”的基本标准。加快学校、医院、公共交通、文化设施、健身场所、电力、通信、燃气、超市、路灯等基本公共服务设施向乡村延伸、向小城镇覆盖，促进公共资源在城乡之间均衡配置、生产要素在城乡之间自由流动，不断让农民和城市居民享受同城化待遇。

（六）以深化改革开放为重点，在增强发展活力上实现新突破

扎实开展“改革开放年”活动，借外力、聚内力、增活力，形成创业、创新、创优的社会环境。一是全力创新，推进重点领域和关键环节改革。深化财税体制改革，贯彻落实中央积极财政政策和税制改革、结构性减税方案，加强财源培育，优化收支结构，强化财政资金监管，逐步推行财政绩效考核，提高财政资金使用效益。进一步完善综合治税信息服务平台，覆盖一市三县。创新区、市、县目标管理方式，将增比进位与干部年度目标、领导班子责任目标考核挂钩。深化投融资体制改革，健全政府重大投资项目核准制和备案制，完善投融资公司法人治理结构，构建可持续发展的政府投融资体系。全力推进国有企业改革重组，实现国有产权集中化管理，健全财务监督、经营预算、产权监管和业绩考核“四大”体系，促进国有企业减数、提质、扩能，发挥国有资本服务政府主导战略和中心任务的能力。深化行政审批制度改革，继续做好市级行政审批事项下放工作，扩大联合审批覆盖面，努力做到审批流程最优、环节最少、效率最高。推进水务一体化改革，实现科学管水、治水、调水、用水。加快推进农村产权制度改革，开展农村土地承包经营权确权登记试点，实施农村土地承包经营权、林权、宅基地使用权作为农业农村发展融资有效担保物试点工作。二是全面放活，推进民营经济超常规发展。加快实施民营经济“三年倍增计划”，全面落实促进民营经济发展政策措施，在放宽、放活、放心、放胆、放手发展民营经济上实现新突破。按照“一视同仁，非禁即入”的原则，将基础设施建设、社会事业发展、生态建设、土地开发等领域全面向民营经济开放。加大对中小企业信贷支持力度，力争城市商业银行60%以上的新增贷款用于支持中小企业、微型企业。扎实开展“3个20万”创业工程，扶持5000户（五年扶持2.5万户）小型微型企业，催生、壮大市场主体，做活民营经济这篇大文章，力争实现民营经济增加值820亿元，增长34%。一市三县要勇立潮头，提升县域经济综合发展实力，在全省县域经济发展中当好排头兵。三是加快开放，积极开展对外交流合作。充分利用国际、国内两种资源、两个市场，促进我市外向型经济向更高水平发展，更有效地发挥全省对外开放制高点的引领作用。积极申报成立碳汇交易市场，加快区域对外贸易中心建设，推进贵阳综合保税区、无水港、小河航天高新技术出口基地等建设，支持25家进出口超过1000万美元企业做大做强，外贸进出口总额达40亿美元。以更大的力度走出去、引进来，实际利用外资3.5亿美元，引进市外到位资金900亿元。

（七）以生态建设为重点，在可持续发展上实现新突破

以推进国家循环经济试点城市、低碳试点城市、节能减排财政政策综合示范城市建设和生态补偿机制试点为契机，加快构建资源节约型、环境友好型社会，让良好的生态服务于经济社会发展。一是实施最严格的生态保护。继续实施以“两湖三库一河”为重点的治水工程、以林业生态建设为重点的绿化工程、以提高空气质量为重点的治污工程，将低碳环保理念深入城市生活和发展之中。启动雅关片区等综合改造项目，拓宽城市污水管网收集和处理系统覆盖面，城区污水处理率达96%以上。加强湿地公园管理和保护，进一步美化花溪国家城市湿地公园景观，申报“两湖三库”国家湖泊生态环境保护试点和国家城市湿地公园。强化建设工地二次扬尘治理，落实施工现场标准化管理，推进文明工地建设。推进农业面源污染治理，完成农村清洁工程20个，建设大中型沼气池30个。建成废旧电子拆解中心和废旧金属市场。二是加快推进生态建设。加强重点水源涵养区、自然保护区生态建设，加大对次生林、坡耕地的改造，把生态建设与产业发展相结合，大力发展茶叶、中药材、经果林等经济效益好的林种，在提高森林覆盖率的同时促进农民增收和农村劳动力转移，提高农民保护生态的积极性，实现生态效益、经济效益、社会效益最大化。巩固退耕还林成果，推进石漠化综合治理植被恢复、天然林资源保护等林业生态工程建设，新增营造林（草）22万亩，森林覆盖率提高1个百分点。加强森林保护，森林火灾受害率、林业有害生物成灾率分别控制在0.6‰、3‰以内。加强城区绿化建设，科学优化黔灵山、东山等城市山体林种结构和生物种群结构。启动实施环城高速公路、二环路等城市主干道沿线景观改造工程，打造一批高品位生态路、景观路，新增绿地40万平方米，形成环、带、线、点四位一体的城市绿化体系。三是大力推进节能减排。建立市级资源综合利用数据库，鼓励企业开展“三废”综合利用，全面推行清洁生产，培育一批资源综合利用龙头企业。加强对重点耗能企业的节能监管，实施强制性清洁生产审核，严查重处无证排污。在城市建设、企业生产、家庭生活中推广运用节能新技术和新产品，抓好联和能源年产300万吨水煤浆项目建设，在十大产业园区、煤化企业、大型房开项目率先推广使用。探索建立梯度用水价格机制，用市场手段调节用水。实施公交车清洁能源应用等14个低碳交通试点项目，增加新能源客运车辆。

（八）以保障和改善民生为重点，在和谐贵阳建设上实现新突破

坚持以人为本、普惠于民，继续实施“十大民生工程”和社会管理创新工程，努力满足人民群众不断增长的物质文化和精神文化需求。市级财政民生支出比上年提高1个百分点。一是推进文化生活大众化。以完善文化阵地、培育时代精神、推进体制改革、发展文化产业为重点，全力促进文化繁荣发展。加强基层文化站（室）建设，重点打造一批历史古镇、历史文化名村、历史文化古迹和历史文化街区，开工建设市档案馆、市工人文化宫等公共文化设施，不断完善公共文化服务基础网络。广泛开展文化进社区、进乡村、进企业活动，深入实施“传统美德弘扬工程”，既丰富群众精神文化生活，又提升市民文明素质。深入挖掘文化资源，开发文化产品，创新文化机制，发展文化产业。开工建设影视文化旅游基地、阳明文化产业园、水田创意文化产业园等项目，支持贵阳日报传媒集团、贵阳广播电视台、贵阳演艺集团、贵阳交响乐团、贵州京剧院提升市场竞争力，促进文化与产业深度融合，加快文化产业多元发展，让更多群众受益。大力倡导诚信文化，切实加强诚信政府、诚信企业、诚信单位、诚信个人为重点的诚信社会建设。二是推进公共服务均等化。深化学区化“五统一”改革，加大对义务教育阶段民办学校的支持力度，积极推进义务教育均衡协调发展，提高进城务工人员子女在公办学校就读的比例；多元化发展学前教育，学前教育入园（班）率达55%；对全市民族贫困乡实行高中阶段免费教育；启动金阳新区普通高中办学试验区建设，扎实推进清镇职业教育聚集区建设；加快幼儿师范专科学校建设并通过国家升格评估，提升贵阳学院、贵阳职业学院等高校办学质量；加强“平安校园”建设，切实保障师生人身安全。加快发展卫生、体育等事业，切实提高全民健康水平。严格执行国家基本药物制度，进一步规范基本药物网上集中采购机制；做好医院改革试点工作，加快市一医一分院、市妇幼保健院综合病房大楼等建设，进一步提升基层医疗卫生网络化服务水平。实施“全民健身计划”，多点建设群众健身活动场所，组团参加全国全省相关体育赛事，支持引进人和中超足球队入驻贵阳。做好奥体中心（一期）开放工作并力争开工建设二期工程。优化利益导向机制，坚持直接控制与间接控制相结合，严格控制人口数量，提高人口素质，继续提升人口计生工作水平。三是推进民生保障一体化。进一步优化创业环境，开展职业技能培训2.2万人，实现城镇新增就业9.95万人，实现农村劳动力就业转移3.2万人，保持城镇“零就业家庭”动态为零；鼓励农民以多种组织形式参与农业农村基础设施建设，增加工资性收入。完善社会保障体系，构建民生安全网。实现城镇职工基本养老、医疗、失业、工伤、生育保险分别扩面5万人、6万人、4万人、4万人、4万人；深入开展城市低收入家庭认定工作，实施城乡低保标准自然增长机制，城乡居民最低生活保障标准提高10%以上。继续实施“农超对接”与价调基金补贴、社会救助保障标准与物价上涨挂钩联动机制，确保主要农副产品市场价格总体平稳，城乡居民生活水平不断提升；加快保障性住房建设，稳定健康发展房地产业，形成合理的多层次住房供给体系，不断满足不同层次需求。整合资源，捆绑实施，连片推进，打好新一轮扶贫脱贫攻坚战，减少新口径贫困人口26万人，10个贫困乡（镇）脱贫摘帽。四是推进社会管理创新化。抓住中央综治委把我市作为西部地区中国特色社会主义大都市样板城市整体推进的机遇，继续加大基层管理体制改革力度，创新社会综合管理服务工作机制，切实抓好流动人口、进城务工人员、留守儿童、空巢老人、失地农民以及吸毒人员、艾滋病患者、刑释解教人员等特殊群体的服务工作，用真心帮助群众，用真情关爱群众，最大限度化解社会矛盾，构建和谐社会。加强社会治安综合治理，强化治安防控体系建设，严厉打击“两抢一盗”、贩毒等犯罪，切实保障人民群众生命财产安全。认真落实安全生产“一岗双责”制度，深入开展煤矿、道路交通、消防、建筑施工、非煤矿山、危险化学品等重点行业（领域）安全生产大检查和隐患大排查，实现生产安全事故起数和死亡人数“双降”。从严加强生产、流通全过程控制，逐步健全覆盖城乡的食品、药品安全监管体系，确保人民群众食品安全、用药安全。在保障和改善民生中，我们还将集中力量为民办好“十件实事”：（1）新建幼儿园9所、改扩建6所，建设农村寄宿制学校20所，新建高中阶段学校2所、改扩建3所，在城区新增初中阶段学校2所。（2）开工建设保障性安居工程1万套（50万平方米），竣工20万平方米。（3）建设10所中心敬老院。（4）对100岁以上、90—99岁、80—89岁老年人分别实行每人每月200元、100元、60元长寿补贴。（5）启动实施10个棚户区、城中村改造，让1.2万户群众受益。（6）实施城乡“四百”工程，以背街小巷、城乡结合部、小区院落综合整治为重点，打造100个文明示范小区；以便民利民为重点，规划建设100个便民市场（社区生鲜直销点）；改造提升100个文明示范村寨；实施100个全民健身工程。（7）实施黔灵湖、小关湖治理工程；实现“两湖三库”一、二级饮用水源保护区内，垃圾收运基础设施建设全覆盖。（8）启动建设餐厨废料（潲水）无害化处理厂。（9）加强交通、城管、治安等信息基础设施建设，实现主城区综合管理视频监控点（探头）、监控报警点全覆盖。（10）引进新能源客运车150辆，新增出租车1500辆以上。老百姓的小事就是政府的大事，我们一定竭尽全力办实办好！

认真贯彻落实党的民族宗教政策，进一步做好民族宗教工作。继续推进外事侨务、对台、移民、气象、史志、老龄、残疾人、双拥等事业发展，支持国防建设和法院、检察院工作，支持工青妇等群团组织做好工作。

四、切实加强政府自身建设

面对新形势、新任务、新挑战，我们将牢记党和人民的重托，不辜负省委、省政府和市委的殷切期望，按照为民、务实、勤奋、清廉的要求，大力提振创先争优士气、激发干事创业热情，不断解放思想、凝聚力量、坚定信心、强化执行，切实加强政府自身建设。

（一）努力建设创新型政府。一是加强学习。时刻把学习作为发展和进步的客观需要，把理论创新作为实践创新的基础，不断提高公务员队伍“看、听、思、说、干”的能力，做到“学以明理、学以养德、学以增智、学以致用、学以创新”。二是解放思想。坚持把党和人民的事业放在首位，敢于担当负责，敢于实践创新，自觉实现由线性思维向复合型思维转变、由局部思维向全局思维转变、由滞后思维向超前思维转变、由封闭思维向开放思维转变，努力使观念更加开明、视野更加开阔、环境更加开放，以思想的大解放促进经济的大发展。三是大胆突破。牢固树立“大发展小困难、小发展大困难、不发展最困难”和“不进则退、慢进也退”的意识，在工作中迎着矛盾上、顶着压力冲，以更大的魄力“闯”、更强的意识“抢”、更猛的劲头“争”、更足的勇气“拼”，敢于担当、大胆突破，推进贵阳经济社会超常规发展。

（二）努力建设服务型政府。一是始终把群众放在心中最高位置。增强民本意识，把群众呼声作为“第一信号”，把群众需要作为“第一选择”，把群众满意作为“第一标准”，深入开展“四帮四促”、干部驻村入企进社区活动，做到面对面引导群众、心贴心关心群众、手拉手帮助群众，全力实现好、维护好、发展好广大人民群众的根本利益。二是始终把服务作为最大的职责。按照“小政府、大服务”的原则，把服务贯穿于政府工作的全过程。深入推进行政审批制度改革，健全完善政务服务体制；坚定不移地推进城市基层管理体制改革，实现重心下沉、服务下移，以更合理的层级、更有效的方式、更务实的作风服务基层、服务群众、服务发展。三是始终把效率作为最直接的目标追求。进一步转变工作作风，能办必办、能快必快、能优必优，切实提高我们的服务质量和办事效率，努力使政府工作在务实高效中推进全市经济社会超常规跨越发展，在务实高效中赢得广大人民群众的信任、拥护和支持。

（三）努力建设法治型政府。一是加强法治宣传教育。不断提高人民群众的法治观念，增强各级公务人员特别是行政执法人员依法履职、依法办事的意识，做到有法可依、有法必依、执法必严、违法必究。二是规范行政决策行为。完善科学民主决策机制。坚持民主集中制，做到重大事项集体研究、集体决策；工作中广纳民意、广集民智，提高政府决策的科学性、民主性和规范性。三是健全完善监督机制。自觉接受人大及其常委会的法律和工作监督，政协和各民主党派、人民团体的民主监督，强化党和政府内部监督，重视新闻舆论和人民群众监督。进一步完善政务公开制度，提高政府工作透明度，做到政府的职能在阳光下运行、政府的工作在监督中推进、政府的公信力在民意中提升！

（四）努力建设廉洁型政府。一是认真落实党风廉政建设责任制和领导干部廉洁从政各项规定。扎实推进惩治和预防腐败体系建设，坚持用制度管权、按制度办事、靠制度管人，从制度上提高拒腐防变的能力。二是加强廉政宣传教育。深入开展正面示范教育和反面警示教育，引导各级公务人员常修为政之德、常怀律己之心、常思贪欲之害，自觉做到稳得住心神、守得住清贫、耐得住寂寞、抵得住诱惑，从思想上筑牢拒腐防变的防线。三是严格执行党的纪律。加大违法违纪案件查处力度，以党风廉政建设和反腐败斗争的实际成效取信于民，以良好的党风促政风带民风，为推动经济社会更好更快发展营造风清气正的社会环境；以良好的政治生态保障经济生态、城镇生态、文化生态、自然生态、社会生态的健康发展。

各位代表，回顾过去，我们负重拼搏、奋力爬高；展望未来，我们豪情满怀、推进跨越。让我们紧密团结在以胡锦涛同志为总书记的党中央周围，在省委、省政府和市委的坚强领导下，牢记“作表率、走前列、做贡献”的光荣使命，在坚持走科学发展路、加快建生态文明市的征程中，燃烧激情、超越自我，用我们的“辛苦指数”提高市民“幸福指数”，用“贵阳速度”展示“贵阳力量”，用“贵阳精神”提升“贵阳形象”，当好全省经济社会发展的“火车头”、黔中经济区崛起的“发动机”，朝着全面建设小康社会的目标奋勇前进，以更加优异的成绩迎接党的十八大和省十一次党代会胜利召开！
